# A retrospective comparison of smart prep and test bolus multi-detector CT pulmonary angiography protocols

**DOI:** 10.1002/jmrs.17

**Published:** 2013-06-07

**Authors:** Tara Suckling, Tony Smith, Warren Reed

**Affiliations:** 1 Medical Imaging Department, Tamworth Rural Referral HospitalTamworth, New South Wales, Australia; 2 University Department of Rural Health, The University of NewcastleTamworth, New South Wales, Australia; 3 Discipline of Medical Radiation Sciences, The University of SydneyLidcombe, Australia

**Keywords:** Bolus tracking, computed tomography pulmonary angiography, pulmonary embolism, test bolus

## Abstract

**Introduction:**

Optimal arterial opacification is crucial in imaging the pulmonary arteries using computed tomography (CT). This poses the challenge of precisely timing data acquisition to coincide with the transit of the contrast bolus through the pulmonary vasculature. The aim of this quality assurance exercise was to investigate if a change in CT pulmonary angiography (CTPA) scanning protocol resulted in improved opacification of the pulmonary arteries. Comparison was made between the smart prep protocol (SPP) and the test bolus protocol (TBP) for opacification in the pulmonary trunk.

**Methods:**

A total of 160 CTPA examinations (80 using each protocol) performed between January 2010 and February 2011 were assessed retrospectively. CT attenuation coefficients were measured in Hounsfield Units (HU) using regions of interest at the level of the pulmonary trunk. The average pixel value, standard deviation (SD), maximum, and minimum were recorded. For each of these variables a mean value was then calculated and compared for these two CTPA protocols.

**Results:**

Minimum opacification of 200 HU was achieved in 98% of the TBP sample but only 90% of the SPP sample. The average CT attenuation over the pulmonary trunk for the SPP was 329 (SD = ±21) HU, whereas for the TBP it was 396 (SD = ±22) HU (*P* = 0.0017). The TBP also recorded higher maximum (*P* = 0.0024) and minimum (*P* = 0.0039) levels of opacification.

**Conclusion:**

This study has found that a TBP resulted in significantly better opacification of the pulmonary trunk than the SPP.

## Introduction

Pulmonary embolism is a common condition with considerable morbidity and mortality.^1^ Because clinical signs or symptoms are often non-specific, diagnosis relies on imaging tests, which include radioisotope ventilation perfusion scanning and, more commonly over recent years, computed tomography pulmonary angiography (CTPA). A CTPA study provides direct visualization of the emboli, as well as additional information relating to alternative diagnoses.^2^

Optimal arterial opacification with contrast medium is crucial in imaging the pulmonary arteries using CT.^3^ The availability of faster scan times has allowed the visualization of the pulmonary vasculature during peak contrast enhancement.^4^ This has posed the challenge of precisely timing the CT data acquisition to coincide with the transit of the contrast bolus through the pulmonary vasculature to achieve optimal opacification. Hartmann et al.^5^ defined optimal opacification to be at least 200 Hounsfield Units (HU).

Using a 64-slice dual-source CT scanner, Henzler et al.^6^ found that bolus tracking resulted in higher mean opacification of the pulmonary trunk (595.2 ± 36.9 HU) compared with using a test bolus (590.3 ± 37.5 HU), although the difference was not statistically significant. Johnson et al.^7^ also found no significant difference between the two different timing techniques, although the results were in relation to ECG-gated CT angiography of the chest. Cademartiri et al.^8^ compared contrast enhancement using both a bolus tracking and a test bolus protocol on a 16-slice multi-detector CT (MDCT) in coronary angiography. They concluded that bolus tracking yielded a more homogenous enhancement, although mean enhancement was higher for the test bolus group (354.7 ± 78 HU) than when bolus tracking was used (305.3 ± 71.4 HU).

As test bolus and bolus tracking protocols are both commonly used in CTPA it is important to investigate which is better for visualization of the arteries and clinical diagnosis. With a view to improving opacification of the pulmonary vessels, Tamworth Rural Referral Hospital (TRRH) in northern NSW changed the CTPA protocol in October 2010 from a bolus tracking protocol (referred to herein as the smart prep protocol or SPP) to a test bolus protocol (TBP). In addition to the change in the timing of data acquisition, the change in protocol incorporated an increase in the injection rate, from 4.0 to 4.5 mL/sec, as described below.

The change was initiated by the consultant radiologist on clinical grounds and not specifically for research purposes. However, it created an opportunity to retrospectively compare pulmonary artery opacification before and after the change was made, as a quality assurance exercise to ensure that the desired outcome was achieved. Therefore, the aim was to investigate which CTPA protocol, SPP or TBP, resulted in the highest opacification of the pulmonary trunk, with the hypothesis being that there was no statistically significant difference in pulmonary artery opacification between the two protocols.

## Method

This study was given institutional approval by the Hunter New England Local Health District as a quality assurance project. TRRH is a 288-bed public hospital located in northern NSW. Its catchment covers an area of 98,000 km^2^, with a population of approximately 178,500 people.^9^ At TRRH, the most commonly used imaging test for diagnosis of a pulmonary embolus is CTPA. The radiology department acquired a 16-slice CT scanner (BrightSpeed, GE Healthcare, Milwaukee, WI) in 2004 and commenced performing CTPA examinations that same year. All examinations in this study were performed on that scanner. TRRH performed 7467 CT examinations for the year 2010, of which 200 were CTPAs. From 2008 to 2010 between 50 and 60 people were diagnosed with a pulmonary embolus each year.

This retrospective study included 160 patients undergoing CTPA during January 2010 and July 2010, with 80 cases being performed with SPP and TBP protocols, respectively. All patients were scanned for a suspected pulmonary embolism. The SPP group consisted of consecutive CTPA patients imaged between January 2010 and July 2010. Training in the TBP took place in October 2010. The TBP was composed of patients imaged between November 2010 and February 2011. CTPA examinations were accessed retrospectively from the picture archive communication system (PACS). The examination type was filtered for “CTPA” or “C/TPA.”

### Image acquisition

CTPA images were acquired in a caudal to cranial direction on suspended inspiration. Acquisition parameters were as follows: 120 kVp; Auto mA (dose modulated); 0.5 sec rotation time; a pitch of 1.375:1; 1.25 mm slice thickness with 20 mm beam collimation. The contrast medium used was Ultravist 370 (Bayer Schering Pharma AG, Germany; Iopamidol, 370 mgI/100 mL) and was administered through an 18 or 20 gauge cannula catheter in an antecubital vein. For all examinations a single chamber power injector (Visimax, Imaxeon P/L, Sydney) was used.

For the SPP, a fixed volume of 100 mL of contrast medium was injected at a rate of 4 mL/sec, whereas for the TBP, the injection rate was 4.5 mL/sec, with 20 mL test bolus injected prior to the main injection. The exact volume of contrast medium used for the TBP image acquisition series was calculated by summing the time to peak and the scan time and multiplying by the injection rate. Thus, the volume varied depending on the patients' blood flow rate, although it was less than 100 mL with shorter injection times in all cases. Neither protocol involved the use of a saline chaser injection, as used in other studies.^6^,^7^

**Figure 1:**
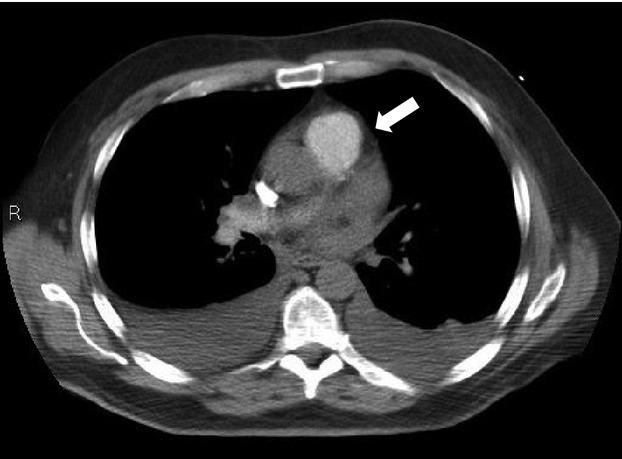
“Blush” of contrast medium seen in the pulmonary trunk (arrowed).

The SPP entailed acquiring non-incremental scans at the level of the pulmonary trunk and/or left and right main pulmonary arteries while contrast medium was being injected. Once the initial “blush” of contrast medium was observed in the pulmonary vessels (Fig. 1), image acquisition was initiated, commencing after a 3 sec delay, during which time the patient was instructed to breathe in and hold their breath.

**Figure 2:**
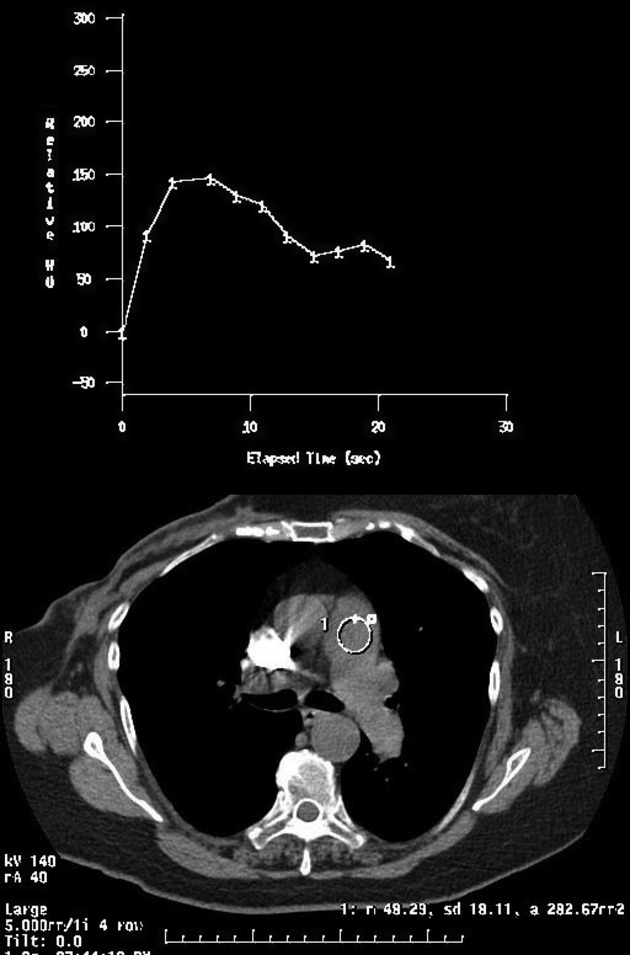
A region of interest (ROI) placed in the pulmonary trunk permits plotting of the Hounsfield Units versus time, demonstrating the time to reach peak enhancement.

For the TBP, the 20 mL test bolus was injected while non-incremental scans were acquired at the level of the pulmonary trunk and/or left and right main pulmonary arteries. Scanning was stopped after the radiographer had seen enhancement and then washout of the contrast medium in the pulmonary vessels. A region of interest (ROI) was then placed within the pulmonary artery outflow tract, and contrast enhancement within the ROI was graphed against time (Fig. 2). The time it took between initiating the injection and peak contrast enhancement was then used as the scan delay time for the CTPA image acquisition series.

### Measurements and analysis

A ROI cursor was placed in the pulmonary trunk to measure the HU, as shown in Figure 2. This was done in the axial plane at the same anatomical location on each examination for both the SPP and TBP images by the lead author (TS). The size of the ROI was kept as constant as possible. The area (in mm^2^), the mean, standard deviation (SD), maximum, minimum, and range (all in HU) were recorded for every examination. Data were entered into a Microsoft Excel™ spread sheet and the average for each ROI variable was calculated for both protocols. Statistical significance was then tested using a two-tailed *t*-test (α = 0.05). No assessment was made of image quality as this was outside the scope of the aim of the investigation.

## Results

**Figure 3:**
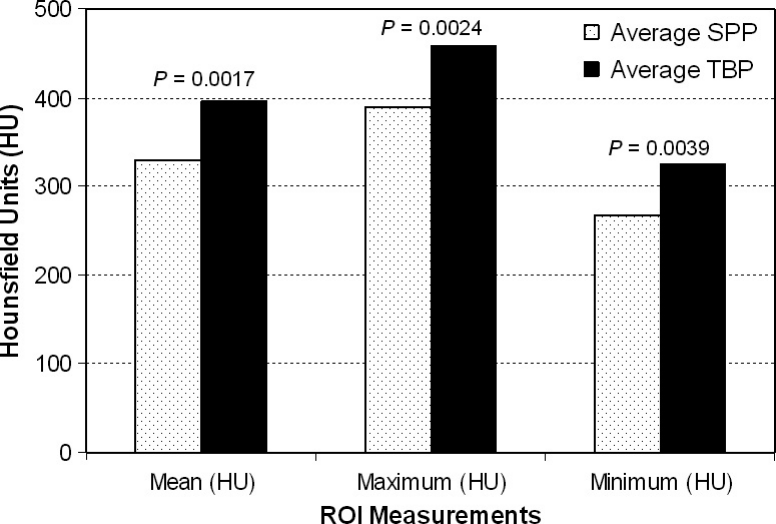
Mean, maximum, and minimum region of interest (ROI) measurements averaged over all the smart prep protocol (SPP) and test bolus protocol (TBP) examinations.

A summary of the findings is given in Table 1. The area of the ROI was marginally greater for the TBP group. Averaged over the 80 examinations in each group, it was found that for the SPP, the mean ROI measurement was 329 (SD = ±21) HU. For the TBP it was significantly higher at 396 (SD = ±22) HU. The TBP also resulted in significantly higher maximum and minimum values. The comparison is shown graphically in Figure 3.

**Table 1:** Average ROI measurements for the SPP cases compared with the TBP cases

ROI	SPP	TBP	*P*-value
Size (mean ± SD) (mm^2^)	199.84 ± 1.19	200.19 ± 0.97	0.0443
Mean (HU)	329.33	396.24	0.0017
Standard deviation (HU)	20.92	22.34	0.1660
Maximum (HU)	389.58	458.79	0.0024
Minimum (HU)	267.04	324.94	0.0039
Range (HU)	122.54	136.38	0.0362

ROI, region of interest; SPP, smart prep protocol; TBP, test bolus protocol; HU, Hounsfield Units.

In terms of optimal opacification of the pulmonary trunk, 98% of the TBP sample had a mean ROI value above 200 HU, whereas the sample SPP only had 90% optimal opacification. The range and SD of the pixel values were both wider for the TBP than for the SPP. This suggests that opacification was less homogenous when using the TBP. The variance in SD, however, was not statistically significant (see Table 1).

## Discussion

The results show that a consistently higher level of opacification of the pulmonary trunk can be achieved using the TBP compared with the SSP, which justifies the change in protocol at TRRH. However, this differs from the findings of previous studies.

Both Johnson et al.^7^ and Henzler et al.^6^ found no significant difference between bolus tracking and a test bolus protocols. Indeed, the Henzler et al.^6^ study found a slightly greater mean opacification of the pulmonary trunk using a bolus tracking protocol, the opposite of the findings of this study. The level of opacification was also much higher in that study, 595.2 ± 36.9 HU for bolus tracking and 590.3 ± 37.5 HU for the test bolus protocol, almost three times the 200 HU minimum recommended level of Hartmann et al.^5^ This may have been due to the higher concentration of contrast medium (Iomeprol 400; Bracco Imaging SpA, Milan, Italy; 400 mgI/mL) and the use of a saline flush, but it does not explain the disparity with the findings of this study.

In both those previous studies, a 64-channel MDCT scanner was used and differences in the scanning parameters compared with GE BrightSpeed may have also contributed to the different findings. Furthermore, the Johnson et al. study was in relation to ECG-gated CT angiography of the chest, not CTPA, and the CTPA in this study was not performed with ECG gating.

Cademartiri et al.^8^ used a 16-slice MDCT to compare opacification of a bolus tracking protocol versus a test bolus protocol in CT coronary angiography, necessitating the use of different scan parameters. They also scanned in the cranio-caudal direction, whereas in this study scanning was in the caudo-cranial direction. In spite of these differences, the findings of this study are consistent with those of Cademartini et al. The levels of opacification were similar and mean enhancement was higher for the test bolus protocol (354.7 HU ± 78) than for bolus tracking (305.3 HU ± 71.4). This suggests that the examination type and scan parameters may not be influential on the results. However, they also concluded that bolus tracking yields more homogenous enhancement, which is not supported in this study, although this may be explained by anatomical differences in the placement of the ROI.

In a review of CT contrast medium administration and scan timing, Bae^10^ argued that the time to peak enhancement should be used to individualize the scan, taking into account individual patient's cardiovascular and contrast medium pharmacokinetic response. The test bolus technique allows visualization of the entire curve of contrast enhancement and demonstrates the exact time that the contrast bolus peaks. Consequently, operator error is reduced when using the TBP, although perhaps not completely eliminated. Theoretically, the timing between the start of the injection and the start of the TBP acquisition should be the same time for both the test bolus and the full bolus of contrast medium. Slight variations in timing, together with minor physiological changes, however, may result in the scan being obtained too early or too late on the enhancement curve, resulting in less than optimal opacification of the pulmonary vasculature. Such sources of error could explain why 2.5% of the TBP study group did not achieve optimal opacification, although this was considerably lower than for the SSP group, where 10% had suboptimal opacification. Some variation may have been due to variation in patient size.

There are some limitations in this study that must be acknowledged. First, as this was a retrospective study, data for some variables were not recorded. For example, knowing the exact contrast medium volume would have allowed a more complete analysis. The longer injection time of the SPP allowed more time for the radiographer to trigger the scan and obtain data during peak enhancement. As pointed out by Bae,^10^ when performing short injections (<10 sec) a test bolus is preferable as there may be insufficient time for bolus tracking to trigger and scan during peak enhancement. In the TBP, increasing the injection rate compresses the bolus, reducing the time to peak enhancement and the injection time. As well as increased vascular enhancement, as demonstrated, it generally decreases the total volume and thus decreases the contrast medium dose to the patient. Again, this information was not available for analysis.

Because this was a retrospective quality assurance exercise, the age, sex, and size of the patient were not recorded. To achieve optimal arterial opacification in larger patients the quantity of contrast medium needs to be increased.^11^ It is not known whether the radiographers compensated for the patient's body habitus when calculating the volume of contrast medium. As the SPP was a standard volume of 100 mL, this could result in variations in opacification depending on the body habitus of patients. It should also be noted that ROI measurements were only recorded for the pulmonary trunk and not for the left and right main pulmonary arteries or their more distal ramifications. While opacification at the level of the pulmonary trunk may be optimal, if the distal branches of the pulmonary vessels are not well filled the examination could still be less than optimum or indeterminate. Finally, another limitation is the use of a 16-slice CT scanner, which has less spatial and temporal resolution compared with more recent 64-slice scanners.

## Conclusion

In CTPA, correct timing of scan acquisition to coincide with the arrival of the contrast medium bolus and opacification of the pulmonary vessels is critical in optimizing the image quality and diagnosis of pulmonary embolism. The purpose of this quality assurance exercise was simply to find whether a protocol change had made an actual, measureable difference to opacification, and this was confirmed. In the cases included in this investigation, not only did the TBP have a higher success rate in achieving optimal opacification in comparison with the SPP but it also resulted in denser opacification of the pulmonary trunk. It supports existing evidence that using a well-planned TBP is more effective than using bolus tracking, such as the SSP.

As this study was completed, the TBP at TRRH has been modified again by the addition of saline chaser bolus injection, suggesting an opportunity for further quality assurance investigation. However, it is recommended that if, in the future, comparisons of MDCT protocols are made, the patient’s weight and contrast volume are recorded so that those variables can be accounted for in the analysis. It would also be of value to evaluate image quality and diagnostic findings.
